# One-stage laparoscopic-assisted resection of gastrojejunocolic fistula after gastrojejunostomy for duodenal ulcer: a case report

**DOI:** 10.1186/1752-1947-5-543

**Published:** 2011-11-05

**Authors:** Masashi Takemura, Genya Hamano, Takayoshi Nishioka, Mamiko Takii, Katsuyuki Mayumi, Takashi Ikebe

**Affiliations:** 1 Department of Surgery, Goshi Hospital 1-8-20, Nagasu-Nishi Dori, Amagasaki City, Hyogo, 660-0807, Japan

## Abstract

**Introduction:**

Gastrojejunocolic fistula is a rare condition after gastrojejunostomy. It was thought to be a late complication related to stomal ulcers as a result of inadequate gastrectomy or incomplete vagotomy. We report a case of gastrojejunocolic fistula after gastrojejunostomy for peptic ulcer treated with one-stage laparoscopic resection.

**Case presentation:**

A 41-year-old Japanese man complained of diarrhea for 10 months, as well as severe weight loss and weakness. After admission, we immediately started intravenous hyperalimentation. On performing colonoscopy and barium swallow, gastrojejunocolic fistula was observed close to the gastrojejunostomy site leading to the transverse colon. After our patient's nutritional status had improved, one-stage surgical intervention was performed laparoscopically. After the operation, our patient recovered uneventfully and his body weight increased by 5 kg within three months.

**Conclusions:**

Modern management of gastrojejunocolic fistula is a one-stage resection because of the possibility of early recovery from malnutrition using parenteral nutritional methods. Today, laparoscopic one-stage en bloc resection may be feasible for patients with gastrojejunocolic fistula due to the development of laparoscopic instruments and procedures. We describe the first case of gastrojejunocolic fistula treated laparoscopically by one-stage resection and review the literature.

## Introduction

Gastrojejunocolic (GJC) fistula is a rare condition after gastrojejunostomy. It was thought to be a late complication related to stomal ulcers as a result of inadequate gastrectomy or incomplete vagotomy [1-3]. In the late 1930s, since patients with GJC fistula were usually malnourished, operative mortality and morbidity were high. Therefore, a two-stage or three-stage procedure was recommended [1]. However, due to recent advances in parenteral nutritional support and intensive care, a one-stage resection can be performed [4].

Currently, surgical treatment for many gastrointestinal diseases can be performed laparoscopically. The aim of this study was to describe the first laparoscopic one-stage resection of a GJC fistula.

## Case presentation

**Figure 1:**
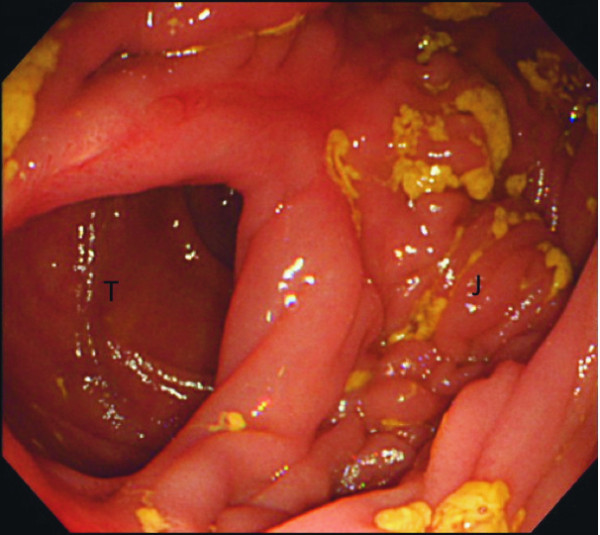
**Colonoscopy showed an abnormal passage between the jejunum and remnant stomach through the fistula (T, transverse colon; J, jejunum)**.

**Figure 2:**
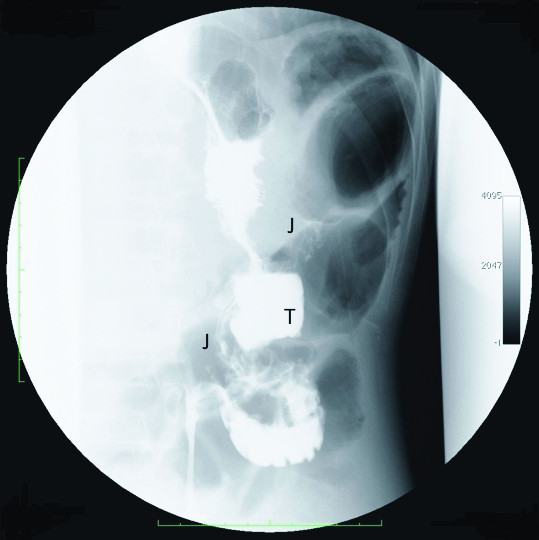
**Barium swallow showed early passage of the contrast media into the colon (T, transverse colon; J, jejunum)**.

A 41-year-old Japanese man was admitted to our hospital complaining of diarrhea immediately after oral intake (10 bowel movements per day for the last 10 months), weight loss (15 kg) and weakness. He reported a partial gastrectomy and gastrojejunostomy due to a duodenal ulcer 18 years prior to his current presentation. On physical examination our patient looked emaciated and dehydrated. Data from laboratory tests performed on admission revealed he had hypoproteinemia and hypoalbuminemia. Parenteral nutrition was started in order to improve our patients' nutritional status. On colonoscopy, the endoscope was able to pass into the remnant stomach through an abnormal fistula that occurred in the transverse colon (Figure 1). Biopsy specimens of the tissue surrounding the fistula were taken and pathology results revealed no malignancies. An upper gastrointestinal endoscopic examination was the performed, revealing a remnant stomach with a Billroth II gastrojejunostomy and a fistula located close to the anastomosis leading to the transverse colon. An upper gastrointestinal series confirmed the existence of an abnormal passage between the remnant stomach and transverse colon (Figure 2).

**Figure 3:**
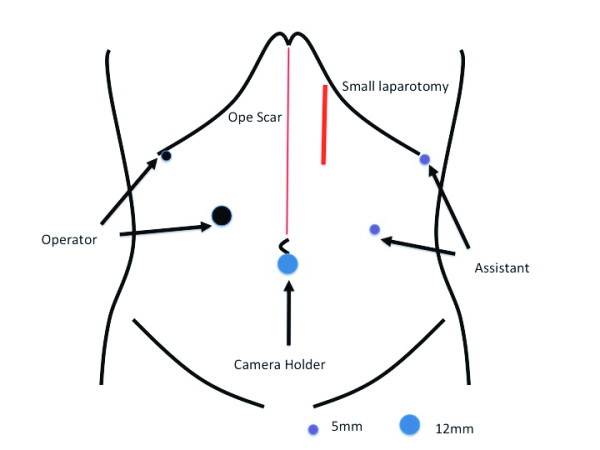
**Trocar placement in our patient**. These locations follow the laparoscopic distal gastrectomy procedures at our institution.

**Figure 4:**
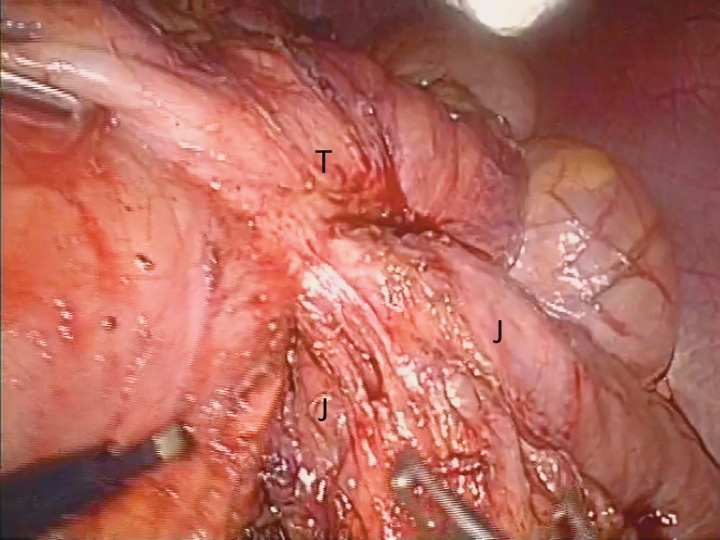
**Laparoscopic view showing moderate adhesion surrounding the remnant stomach**. Retrocolic gastrojejunostomy was identified.

**Figure 5:**
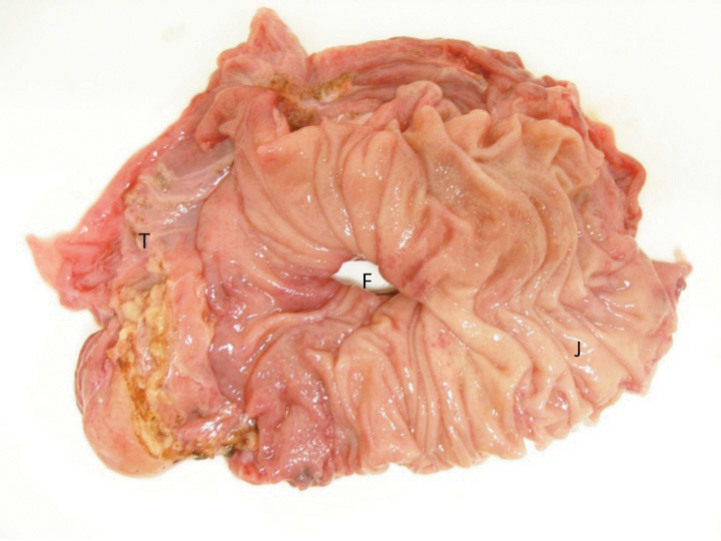
**Macroscopic findings of the en bloc resection specimen of the fistula (F) measuring 2 cm in diameter (T, transverse colon; J, jejunum)**.

When our patient's nutritional status had improved, a laparoscopic surgical resection was performed successfully. Trocars were placed according to laparoscopy-assisted distal gastrectomy (Figure 3). Intra-operatively, moderate adhesions between the remnant stomach, transverse colon, and proximal jejunum were identified, as well as a retrocolic gastrojejunostomy (Figure 4). A radical one-stage laparoscopic en bloc resection was performed, involving partial gastrectomy, segmental resection of the jejunum with conversion into a Roux-en-Y anastomosis and segmental resection of the transverse colon with end-to-end colocolostomy through a small laparotomy (5 cm). The operation duration was 260 minutes and the blood loss was 50 g. Pathology results revealed no evidence of malignant cells within the fistula (Figure 5). Our patient's post-operative course was uneventful and oral nutrition was resumed on the seventh post-operative day. Three months after the operation our patient is well and his body weight has increased by 5 kg.

## Discussion

Gastrojejunocolic fistula is an uncommon late complication after gastrojejunostomy for peptic ulcer or malignant gastrointestinal diseases [1,2]. This fistula is thought to occur due to inadequate gastrectomy, simple gastroenterostomy, or inadequate vagotomy. In the past, this complication was associated with high mortality because of the poor nutritional status of patients with a GJC fistula. Divided operations have been indicated in order to decrease post-operative mortality [1,5]. Recently, the incidence of such fistulas has been decreased dramatically due to conservative treatment of peptic ulcers with H2 receptor antagonists, proton pump inhibitors and eradication regimens for *Helicobacter pylori *and the limitation of surgical treatment in extreme cases [6,7]. However, the fistula can develop one to 20 years after gastrectomy [1]. Therefore, this condition is still important and the contribution of previous surgery should not be overlooked. The typical symptoms of GJC fistula are diarrhea and weight loss. Marshall and Knud-Hansen reported that both these symptoms were present in 80% and 82% of patients [2]. Other less common symptoms in GJC fistula are fecal vomiting or fecal breath, and weakness. In our case, fecal vomiting was not noted, but weight loss and immediate diarrhea after oral intake suggested GJC fistula. Furthermore, the possible cause of the fistula formation in our patient's case was inadequate gastrectomy.

A barium enema or endoscopy are essential for the correct diagnosis of GJC fistula [6]. Thoeny *et al*. reported that a barium enema is useful in making a diagnosis of GJC fistula with significantly higher sensitivity than upper gastrointestinal series [8]. Recently, endoscopy and colonoscopy have been recommended for the diagnosis of GJC fistula to exclude other gastrointestinal diseases. Nussinson *et al*. reported the usefulness of endoscopy together with colonoscopy in the diagnosis of GJC fistula [9]. However, in some reported cases that examined the efficacy of colonoscopy, fistula was not detected [7]. Thus, negative findings from colonoscopy are insufficient to the rule out a diagnosis of GJC fistula. In our patient's case, endoscopy and colonoscopy revealed the fistula and were useful to exclude other malignant gastrointestinal diseases.

Surgery is one of the curative treatments for GJC fistula. In the late 1930s, a three-stage procedure consisting of colostomy, resection of the fistula, and closure of the colostomy was defined [4]. The disadvantage of this procedure is that three major surgical procedures are required for each patient. Lahey proposed a two-stage procedure including a proximal defunctionalized ileosigmoidostomy followed by resection of the fistula, subtotal gastrectomy, and colectomy [10]. Lahey's procedure significantly reduced mortality and morbidity in patients with GJC fistula, and has been widely accepted as the treatment of choice. Recently, because of the development of parenteral or enteral nutrition support and improvements in intensive care, one-stage en bloc resection is accepted as the procedure of choice and mortality and morbidity due to GJC fistula have been decreased [4,6,8].

Today, surgical intervention for many gastrointestinal diseases can be performed laparoscopically. However, laparoscopic resection in a case of GJC fistula has not been reported. In our patient's case, moderate adhesion was seen in the area of the remnant stomach laparoscopically. All abrasion procedures were performed laparoscopically, and en bloc resection and reconstruction were performed via a small laparotomy (5 cm). Laparoscopic procedures are demanding for patients with a history of major abdominal surgery. However, we think that laparoscopic surgery for patients with GJC fistula, many of whom are malnourished, may be useful because it is less invasive than open surgery.

## Conclusions

Gastrojejunocolic fistula has been considered a rare complication after gastrectomy or gastrojejunostomy. Endoscopy and colonoscopy are useful diagnostic tools for GJC fistula, but negative findings from endoscopy do not exclude the presence of fistula. Modern management of GJC fistula is via a one-stage resection. Today, laparoscopic-assisted one-stage en bloc resection may be feasible for patients with GJC fistula.

## Consent

Written informed consent was obtained from the patient for publication of this case report and any accompanying images. A copy of the written consent is available for review by the Editor-in-Chief of this journal.

## Competing interests

The authors declare that they have no competing interests.

## Authors' contributions

MT, KM and TI collected the data, drafted and wrote the manuscript. GH, TN and MT contributed to our patient's post-operative management and approved the final manuscript. All authors read and approved the final manuscript.
